# Chylous fistula after right breast fibroadenoma resection: a case report

**DOI:** 10.3389/fsurg.2023.1269301

**Published:** 2024-01-04

**Authors:** Haoxi Liu, Liping Liu, Xinhua Yang

**Affiliations:** Department of Breast and Thyroid Surgery, Guiqian International Hospital, Guiyang, China

**Keywords:** chylous fistula, breast, fibroadenoma, surgery, case report

## Abstract

Chylous fistula is a common postoperative complication for the head and neck surgery, thoracic and upper gastrointestinal surgery, but it rarely happens after breast surgery. There are few reports of chylous fistula after breast benign tumor resection according to literature retrieval. To our acknowledgement, this is the first case report of chylous fistula after breast fibroadenoma resection.

## Introduction

Chylous fistula usually occurs after radical neck dissection and thoracic surgery, but it seldom happens after breast surgery. Its incidence is 0.5%–8.3% in patients receiving radical neck dissection (1), 0.36%–0.84% in patients receiving breast cancer surgery (2). In this article, we reported a case of chylous fistula after right breast fibroadenoma resection without axillary dissection, and summarized the etiology, prevention and treatment strategy for the patient.

## Case report

**Figure 1:**
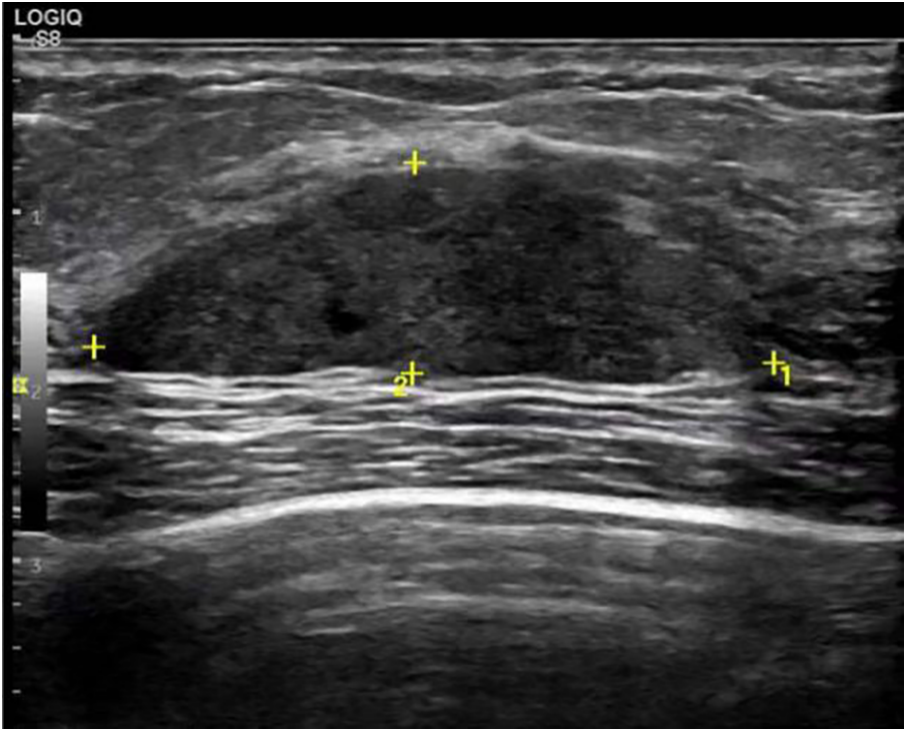
Color Doppler ultrasound showed a solid phyma, with regular shape and clear boundary.

**Figure 2:**
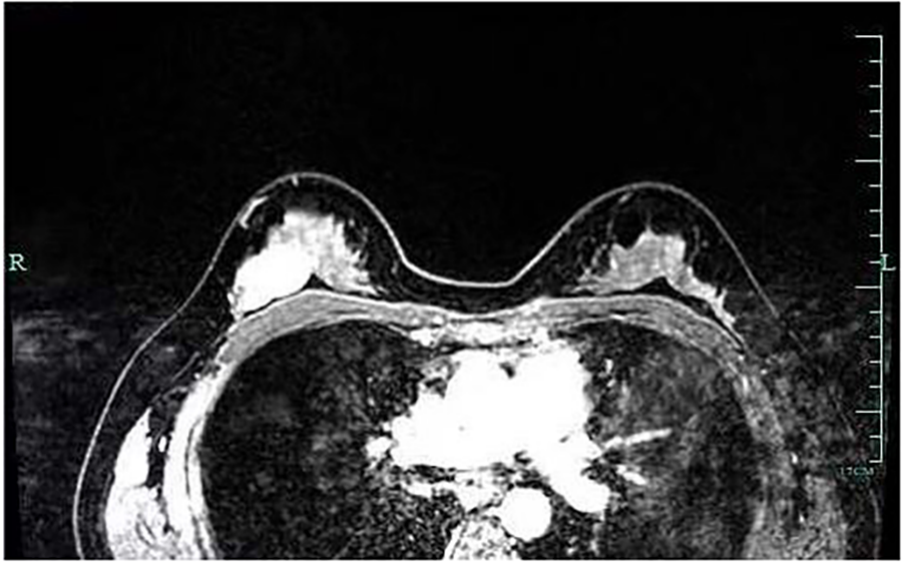
Breast MRI indicated a nodular lesion of abnormal enhancement, with clear boundary and slightly irregular shape.

**Figure 3:**
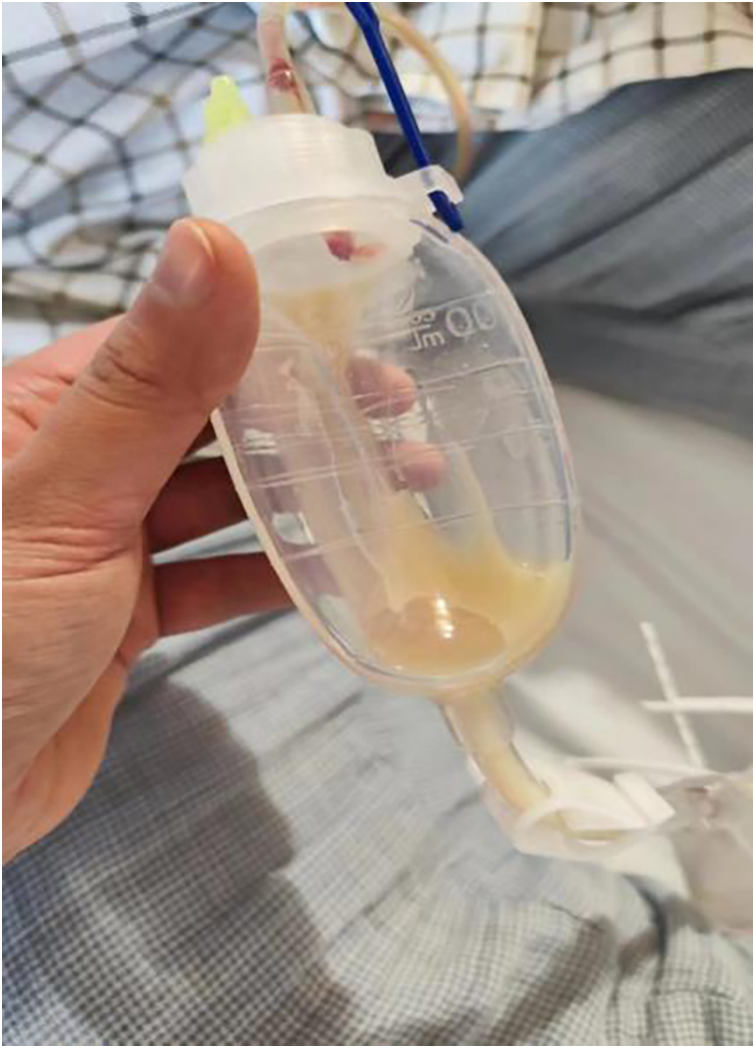
On postoperative day 2, there was milky fluid in the drainage tube, suggesting the existence of chylous fistula.

**Figure 4:**
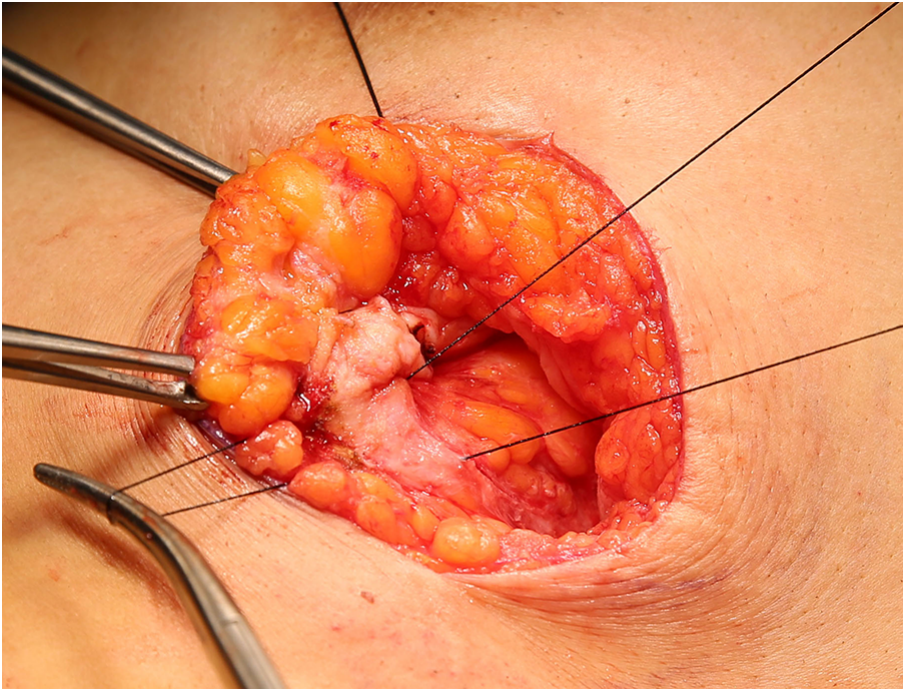
During operation, we performed segmental ligation on of fresh granulation tissue after removing necrotic tissue.

**Figure 5:**
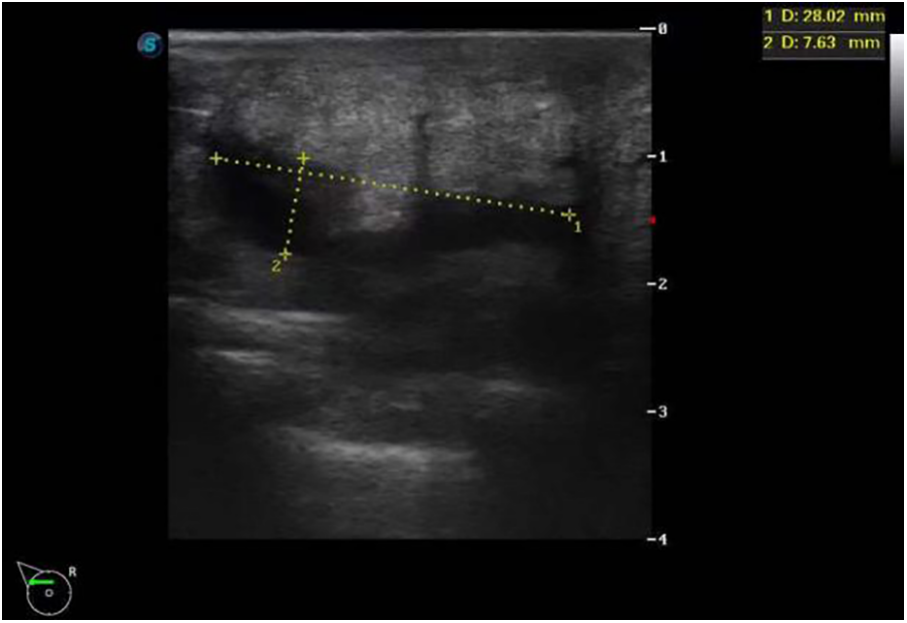
Color Doppler ultrasound indicated a small amount of fluid accumulated in the surgical area.

A 29-year-old woman was admitted to Guiqian International Hospital because of a palpable lump on the right breast for 3 months. She had no special medical history. High-frequency ultrasound showed a 3.90 cm × 1.21 cm × 4.22 cm solid phyma in the upper outer quadrant of the right breast, with clear boundary and regular shape, and no significant enlargement of axillary lymph nodes (Figure 1). The magnetic resonance imaging (MRI) of the breast indicated a 3.65 cm × 1.10 cm × 4.01 cm nodular lesion of abnormal enhancement at approximately 10 o'clock in the right breast, with clear boundary and slightly irregular shape (Figure 2). Breast phyma resection was applied under local infiltration anesthesia. Due to the large size of the lump and the wide range of residual cavity in the surgical area, closed suction drainage was placed in the surgical area to prevent fluid accumulation. Postoperative pathology confirmed the diagnosis of breast fibroadenoma, adenosis and metaplasia of the sweat gland. On postoperative day 2, 90 ml of milky liquid was observed in the closed suction drainage (Figure 3). Subsequently, a serologic test for celiac disease proved to be positive. Therefore, conservative treatment was given, including compressive bandage and a low-fat diet. Meanwhile, a new method of dressing change was used, i.e., repeated rinsing with hydrogen peroxide and physiological saline in the surgical area, and multiple local injections of hypertonic syrup and tuberculin pure protein derivatives from the drainage tube with a syringe, in order to promote local adhesion and reduce the risk of fluid accumulation. However, the outcome was not satisfactory and daily drainage was 50–120 ml/day for 11 days. After obtaining the consent of the patient, we performed a reoperation to explore the surgical area. During operation, we removed necrotic tissues, and carefully searched for the leakage, but unfortunately we couldn't find it. Therefore, we performed segmental ligation on fresh granulation tissue after removing necrotic tissue to achieve the goal of ligating potential leakage sites and further promote fibrosis of the tissue (Figure 4). Finally, the incision was closed, with one drainage tube replaced. After reoperation, daily drainage dropped to 30–75 ml/day for 10 days, suggesting that chylous leakage continued but with decreased volume. The color Doppler ultrasound showed a small amount of fluid accumulated in the surgical area (Figure 5) and then the drain tube was removed on postoperative day 11. After extubation, there was a little fluid in the surgical area, which was relieved by compressive bandage and a low-fat diet. On postoperative day 13, the patient was discharged uneventfully.

## Discussion

Chylous fistula is a rare complication that, if manifested, can exacerbate the patients' pain and psychological distress, prolong hospital stay, and increase financial burden. Its diagnosis and treatment is challenging in clinic. The literature shows that chylous fistula is primarily observed after axillary dissection for left breast cancer (3–5), whereas chylous fistula after right axillary dissection is infrequent (2, 6, 7). A few cases of chylous fistula following axillary sentinel lymph node biopsy have been reported (6, 8). However, a comprehensive literature review indicates that chylous fistula is generally associated with breast surgery involving axillary procedures, with only a few isolated cases not involving such procedures (9–11). To the best of our knowledge, no case of chylous fistula following benign breast tumor resection without axillary dissection has been reported yet.

In anatomy, the thoracic duct starts from the cisterna chyli, enters the thoracic cavity through the aortic hiatus, ascends to the level of the fifth thoracic vertebra, inclines to the left, and ascends along the left front of the spine, to receive the lymph of the lower limbs, pelvis, abdomen, left upper limb, left chest, head and neck. The right lymphatic duct drains lymph from the right upper limb, right chest, head and neck. In some cases, the thoracic duct bifurcates into right and left branches during its course through the thoracic region: the left branch proceeds in the conventional manner, while the right branch terminates by joining the right subclavian vein, in conjunction with the right lymphatic duct (12). A documented case exists in the literature wherein the thoracic duct deviated from its usual course and persisted on the right side, ultimately terminated into the right internal jugular vein (13). The lateral and upper lymphatic vessels of the breast converge to form the subclavian trunk and neck trunk, and the right side flows into the right lymphatic duct and the left side into the thoracic duct. Based on their relationship with the breast parenchyma, the lymphatic vessels draining from the breast to the axilla can be classified into two groups: the deep system and the superficial system, each with its own independent lymphatic drainage pathways to the armpits. Suami et al. (14) reported that in the breast region, most of the lymph collectors run between the dermis and the breast tissue and there are also perforating lymphatic vessels in the breast gland itself. The lymphatic plexus under the areola, the dermis, subcutaneous lymphatic vessels, and lymphatic vessels in the gland compose a widely interconnected lymphatic network.

The underlying mechanism of axillary chylous fistula remains unclear yet. According to some studies, chylous fistula in the axilla can be caused by the injury of the abnormal branch of thoracic duct draining the left axilla (5), or the the subclavian duct in the right axilla (15). Langford et al.'s (12) study showed that the path of the thoracic duct from the tracheo-oesophageal groove to the venous angle remained constant, and in all 24 cadavers the duct approached the venous angle from behind and terminated within 1 cm of the venous angle, which was also previously reported by Parsons and Sargent (16) and by Van Pernis (17). The usual termination of the thoracic duct is single-sited, and the most common site is the jugulo-subclavian angle (12, 18). Therefore, we concluded that the injury to the thoracic duct or its branches may not result in chylous fistula, but injury to the subclavian duct or its tributary during axillary dissection can cause chylous fistula, which is consistent with the viewpoint of Singh et al. (15).

In this case, we only removed the breast fibroadenoma, without axillary lymph node dissection. Her chylorrhea may be attributed to an altered thoracic duct or cervical lymphatic duct injury. According to our experience of neck surgery, if the thoracic duct, the left cervical lymphatic duct, or its main branches, are directly injured intraoperatively, chylous leakage volume will be higher than 1,000 ml per day. The maximum daily drainage of this patient was 120 ml, so it suggested that the chylorrhea was caused by damage to the secondary branch of the left cervical lymphatic duct through the breast tissue, rather than the left cervical lymphatic duct or this primary branch.

Most of chylous fistula patients are treated by conservative management, consisting of a fat-free diet allowing only medium-chain fatty acids, adequate rest, closed drainage, and pressure dressings (15, 19). Although there is growing evidence that nutrition plays a role in the management of chylous fistula, it is hard to determine which dietary is more effective (20). Refractory cases of lymphatic and chylous fistula have been treated with octreotide (a long-acting synthetic analogue of somatostatin) and orlistat (a pancreatic lipase inhibitor) (21, 22).

Surgical intervention is preferred for patients with high output chylous fistula,, leakage persists for more than 2 weeks, daily drainage volume is continuously over 1 L for 1 week, or patients present metabolic complications (9, 15, 19).

Since this patient's chylorrhage had persisted for nearly 2 weeks, we scheduled a reoperation to find the damaged lymphatic vessels, but failed during operation. Unlike chylous leakage in the neck surgery, fluid volume was extremely limited. Finally, we removed the suspected necrotic tissue and re-sutured the mammary gland tissue to reduce the residual cavity.

Meanwhile, we adopted a new method of dressing change, to be specific, multiple local injections of hypertonic syrup and tuberculin pure protein were given to emanate aseptic inflammatory reactions, cause local adhesion and promote the healing. But the outcome was not satisfactory. Fortunately, the patient's drainage was slowly decreased, and she was eventually discharged on postoperative day 13.

## Conclusion

We effectively managed chylous fistula following benign breast tumor resection through a carefully planned regimen. Timely diagnosis and surgical interventions are crucial in the treatment, especially for the cases that chylous output remains low but persists for a long time (e.g., 2 weeks). Furthermore, breast surgeons should have an in-depth understanding of breast lymphatic anatomy and beware of potential anatomical variations, which is essential for the identification and appropriate management of this complication.

## Data Availability

The original contributions presented in the study are included in the article/Supplementary Material, further inquiries can be directed to the corresponding author.
